# Structural Stability of Diffusion Barriers in Cu/Ru/MgO/Ta/Si

**DOI:** 10.3390/nano5041840

**Published:** 2015-11-03

**Authors:** Shu-Huei Hsieh, Wen Jauh Chen, Chu-Mo Chien

**Affiliations:** 1 Department of Materials Science and Engineering, National Formosa University, 64 Wunhua Road, Huwei, Yunlin 632, Taiwan; E-Mails: shhsieh@nfu.edu.tw (S.-H.H.); tienmcyt@gmail.com (C.-M.C.); 2 Graduate School of Materials Science, National Yunlin University of Science and Technology, 123 University Road, Section 3, Douliou, Yunlin 640, Taiwan

**Keywords:** diffusion barrier, ultra-thin film, Cu metallization, Cu_3_Si, Ru/MgO/Ta

## Abstract

Various structures of Cu (50 nm)/Ru (2 nm)/MgO (0.5–3 nm)/Ta (2 nm)/Si were prepared by sputtering and electroplating techniques, in which the ultra-thin trilayer of Ru (2 nm)/MgO (0.5–3 nm)/Ta (2 nm) is used as the diffusion barrier against the interdiffusion between Cu film and Si substrate. The various structures of Cu/Ru/MgO/Ta/Si were characterized by four-point probes for their sheet resistances, by X-ray diffractometers for their crystal structures, by scanning electron microscopes for their surface morphologies, and by transmission electron microscopes for their cross-section and high resolution views. The results showed that the ultra-thin tri-layer of Ru (2 nm)/MgO (0.5–3 nm)/Ta (2 nm) is an effective diffusion barrier against the interdiffusion between Cu film and Si substrate. The MgO, and Ta layers as deposited are amorphous. The mechanism for the failure of the diffusion barrier is that the Ru layer first became discontinuous at a high temperature and the Ta layer sequentially become discontinuous at a higher temperature, the Cu atoms then diffuse through the MgO layer and to the substrate at the discontinuities, and the Cu_3_Si phases finally form. The maximum temperature at which the structures of Cu (50 nm)/Ru (2 nm)/MgO (0.5–3 nm)/Ta (2 nm)/Si are annealed and still have low sheet resistance is from 550 to 750 °C for the annealing time of 5 min and from 500 to 700 °C for the annealing time of 30 min.

## 1. Introduction

Regarding diffusion barrier layers for copper metallization in VLSI, it is quite important to find an appropriate material as an effective diffusion barrier between copper and silicon. The diffusion barrier should possess some features which include [1,2]: thermodynamically stable when in contact with Cu and Si, amorphous structure to prevent diffusion along the grain boundaries, good adhesion between the barrier and the surrounding material, having an electrochemical potential similar to that of Cu and Si to avoid the formation of galvanic cells, low stress in the barrier material, low resistance contact with Cu and Si, and reasonable thermal conductivity.

Up to now, there are many materials which have been studied and developed to be used as diffusion barrier against the interdiffusion between Cu and Si, such as W, Ta, Zr, TaNi, TiW, TaN, TiN, TaSiNi, CoW, Ta-Si-N, W-B-N, Mo-V-N, Al-Cr-Ta, Ti-Zr-N, Al-Mo-Nb-Si-Ta-Ti-V-Zr-N, and so on [3,4]. Furthermore, the thickness of diffusion barrier is decreased with the decrease of integrated circuit size. For examples, a 13 nm-thick Ta-Rh film as a diffusion barrier was prepared and studied by Dalili *et al.* [5], a 4 nm-thick Ru film as a seed layer for copper metallization by Yeo *et al.* [6], and 15nm-thick Ru-N and Ru-Ta-N films have been deposited and applied as diffusion barrier [6].

Magnesia (MgO) is a well-known refractory oxide which has a melting point of 2800 °C, an optical band gap of 7.2 eV, an optical absorption coefficient/cm (7 μm) of 0.05, and a dielectric constant of 9.8 [7]. MgO thin films have been widely used as chemically stable buffer layers for the deposition of high *T*_c_ superconducting films, (*T*_c_ being a critical temperature for a superconductor), perovskite-type ferroelectric films, and a protecting layer of dielectrics [8]. MgO thin films are also used to catalyze the H_2_ and O_2_ exchange reaction [9]. It has been shown that Mn does not appear to diffuse into the MgO barrier in annealed a CoFe/MgO/IrMn magnetic tunnel junction with an MgO tunnel barrier [10]. The electrical conductivity of MgO film is quite low. However, the ultra-thin MgO film has good electric conductivity due to the dissipation of electrical charge via the metallic substrate [11] and due to the tunneling effect [12].

Copper interconnection has been widely used in integrated semiconductor circuit technology because of its low electrical conductivity and superior resistance to electromigration [13]. However, the interaction between Si and Cu is strong and detrimental to the electrical performance of Si [14,15,16]. It is necessary to implement a diffusion barrier between Cu and Si to prevent devices from deteriorating interdiffusion. There are various materials such as refractory metals, nitrides, carbides, oxides, and borides have been recognized as diffusion barriers [17,18,19,20,21]. However, the 2013 International Technology Roadmap for Semiconductors demands an ultrathin diffusion barrier (<5 nm) for the future metal interconnection [22]. As for the current, TaN/Ta encounters scaling difficulty at the 22 nm node. Therefore, A Cu seed layer is necessary for electroplating Cu interconnector on Ta and TaN diffusion barriers.

Ruthenium (Ru) is a noble metal with a high melting point of 2334 °C and Cu can be electroplated directly on to Ru without a seed layer. However, pure Ru may not be a good diffusion barrier, because the 15 nm and 5 nm thick Ru thin film is an effective barrier up to 450 and 300 °C annealing, respectively [23,24]. Ta has high activation energy for both lattice and grain boundary self-diffusion due to its high melting point (3020 °C). Ta is also compatible with the current Cu low-k interconnect system because Ta/TaN layers are employed in a traditional Cu-seed/Ta/TaN trilayer liner. However, a 50 nm thick Ta thin film prevents Cu-silicon interaction up to 550 °C for 30 min in flowing purified He [25]. In order to improve the barrier performance of ultrathin Ru and Ta films, it is necessary to interpose an ultrathin MgO layer between Ru and Ta films. Thus, in this study, the diffusion-barrier performance of Ru/MgO/Ta ultrathin films is explored using Cu/Ru/MgO/Ta/Si stacking structures.

In the present work, Cu (50 nm)/Ru (2 nm)/MgO (0.5–3 nm)/Ta (2 nm)/Si structures were prepared, in which the ultra-thin multilayer of Ru (2 nm)/MgO (0.5–3 nm)/Ta (2 nm) with a total thickness of 4.5–7 nm was used as a new diffusion barrier against the interdiffusion between Cu and Si. The effect of the thickness of ultra-thin MgO films on the failure behavior of Ru/MgO/Ta diffusion barrier between electroplating Cu and Si substrate annealed in a low vacuum was investigated.

## 2. Results and Discussion

### 2.1. Cu (50 nm)/Ru (2 nm)/MgO (3 nm)/Ta (2 nm)/Si

**Figure 1:**
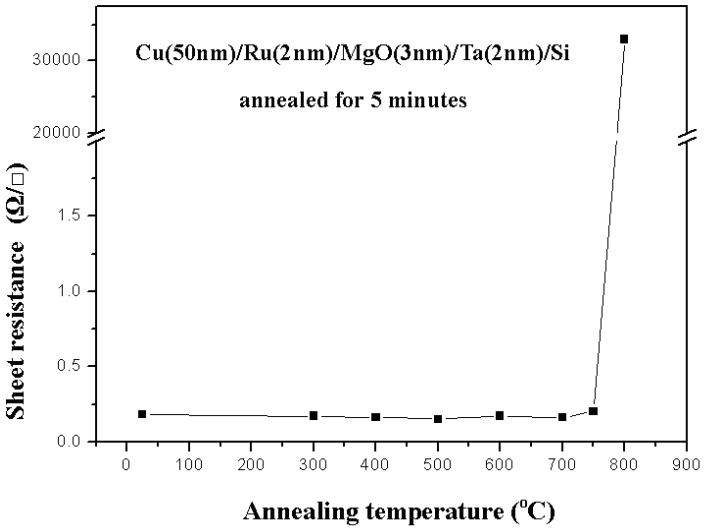
Relation between sheet resistances and annealing temperatures for Cu (50 nm)/Ru (2 nm)/MgO (3 nm)/Ta (2 nm)/Si structure annealed for 5 min.

**Figure 2:**
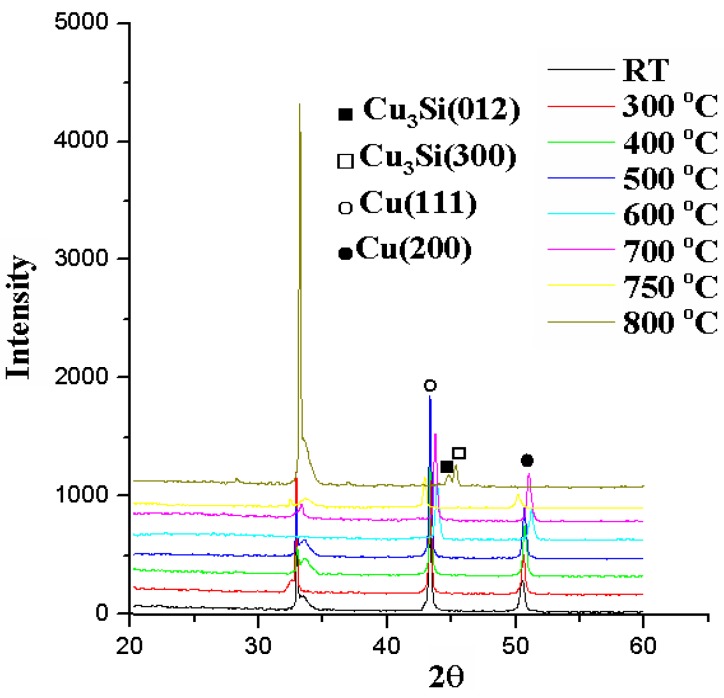
X-ray diffraction patterns for the MgO (3 nm) sample annealed for 5 min at temperatures from room temperature to 800 °C.

Figure 1 shows the relation between sheet resistance and annealing temperature for a Cu (50 nm)/Ru (2 nm)/MgO (3 nm)/Ta (2 nm)/Si structure annealed for 5 min. The sheet resistances are about 0.25 ohm/square (Ω/ϒ) when the annealing temperature is not higher than 750 °C. However when the annealing temperature is 800 °C, the sheet resistance increases abruptly up to 30,000 ohm/square (Ω/ϒ). Figure 2 shows the X-ray diffraction patterns for the Cu (50 nm)/Ru (2 nm)/MgO (3 nm)/Ta (2 nm)/Si structure annealed for 5 min at temperatures from 300 to 800 °C. The occurrence of Cu_3_Si phase in the structure can explain why the sheet resistance of the structure annealed at 800 °C for 5 min increases abruptly up to that more than 30,000 ohm/square (Ω/ϒ). XRD analysis of the samples after annealing to 750 °C shows almost exactly the same pattern as that of the as-deposited sample except for some changes in relative intensities. Based on these XRD spectra, only copper is present up to 750 °C. At 800 °C, the formation of copper silicide, Cu_3_Si, take place according to the XRD. As mention above, the Cu_3_Si formation is not observed at the annealing temperature of 750 °C. It is well know that the Cu_3_Si phase has been formed by solid-state reaction at a temperature of 200 °C, which is much lower than the annealing temperature used in this study. Thus, Ru (2 nm)/MgO (3 nm)/Ta (2 nm), as a barrier material, is useful to block the diffusion of Cu into Si.

**Figure 3:**
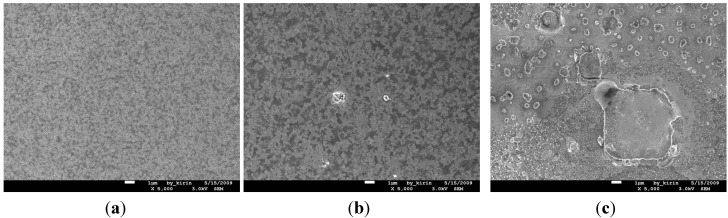
Scanning electron microscope (SEM) views for the surface morphologies of MgO (3 nm) sample annealed for 5 min at temperatures of 700 °C (**a**); 750 °C (**b**); and 800 °C (**c**); respectively.

Figure 3a–c show the SEM images for the surface morphologies of Cu (50 nm)/Ru (2 nm)/MgO (3 nm)/Ta (2 nm)/Si structure annealed for 5 min at 700, 750, and 800 °C, respectively. The surface of the structure annealed at 700 °C looks relatively smooth, as shown in Figure 3a. When annealed at 750 °C, the surface of the structure looks relatively rough, as shown in Figure 3b, which may be ascribed to the agglomeration of the Cu layer. In Figure 3c there are many particles with a size of about 0.1–10 nm present at the surface, which are Cu_3_Si phase confirmed by the X-ray diffraction, as shown in Figure 2.

**Figure 4:**
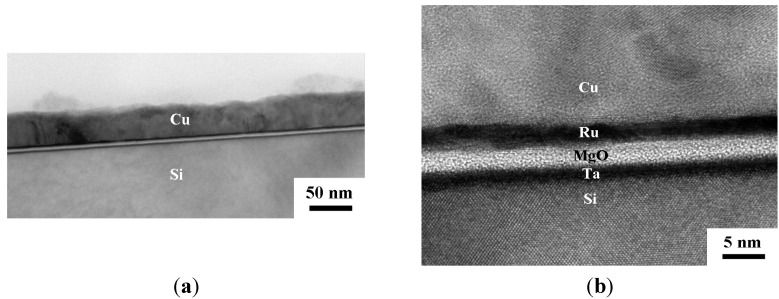
Transmission electron microscope (TEM) cross section views for the MgO (3 nm) sample as deposited at a relatively low magnification (**a**); and at a relatively high magnification (500,000×) (**b**).

**Figure 5:**
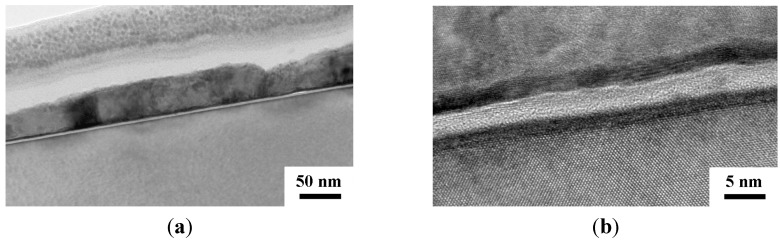
TEM cross section views for the structures of MgO (3 nm) sample annealed for 5 min at 500 °C at a relatively low magnification (**a**); and at a relatively high magnification (**b**).

**Figure 6:**
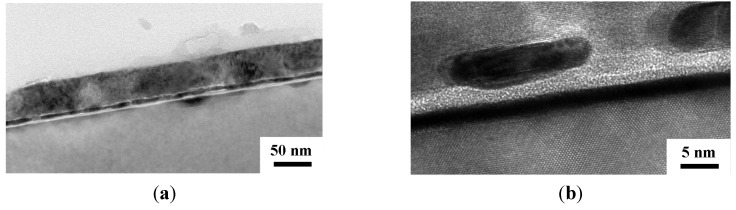
TEM cross section views for the structures of Cu (50 nm)/Ru (2 nm)/MgO (3 nm)/Ta (2 nm)/Si annealed for 5 min at 750 °C at a relatively low magnification (**a**); and at a relatively high magnification (**b**).

**Figure 7:**
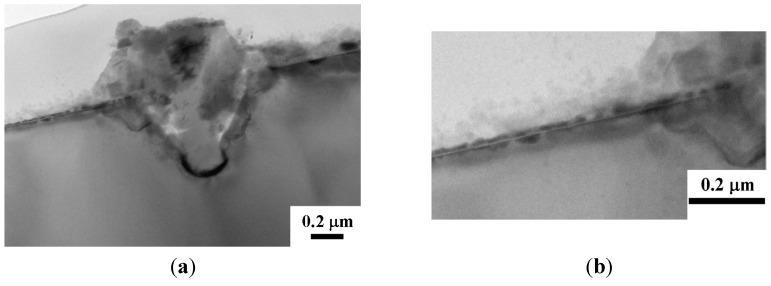
(**a**) TEM cross section views for the MgO (3 nm) sample annealed for 5 min at 800 °C; (**b**) Enlarged view of the left part of (**a**).

The TEM cross section views for the Cu (50 nm)/Ru (2 nm)/MgO (3 nm)/Ta (2 nm)/Si structure annealed for 5 min at room temperature, 500, 750, and 800 °C are shown in Figure 4, Figure 5, Figure 6 and Figure 7, respectively. The as-deposited Ru layer, MgO layer, and Ta layer in the structure are quite flat and the MgO layer and Ta layers are amorphous, as shown in Figure 4a,b. After annealing at 500 °C for 5 min, the surface of the Ru layer, MgO layer, and Ta layer are still flat and the MgO layer and Ta layer are still amorphous, as shown in Figure 5b. After annealing at 750 °C for 5 min, the MgO layer and Ta layer are still amorphous and continuous, but the Ru layer became discontinuous and there is some Cu diffusion at the discontinuities, as shown in Figure 6. Figure 7a shows glom Cu_3_Si particle that formed in the structure after annealing for 5 min at 800 °C. An enlarged view of the left part of Figure 7a is shown in Figure 7b, which clearly shows that both Ru and Ta layers are discontinous, but the MgO layer seems to be still continuous. Therefore, it can be speculated that the Ru layer first became discontinuous at 750 °C, the Ta layer sequentially became discontinuous at 800 °C, the Cu atoms then can diffuse through the MgO layer and to the substrate at the discontinuities, and the Cu_3_Si phase finally form.

### 2.2. Cu (50 nm)/Ru (2 nm)/MgO (2 nm)/Ta (2 nm)/Si

**Figure 8:**
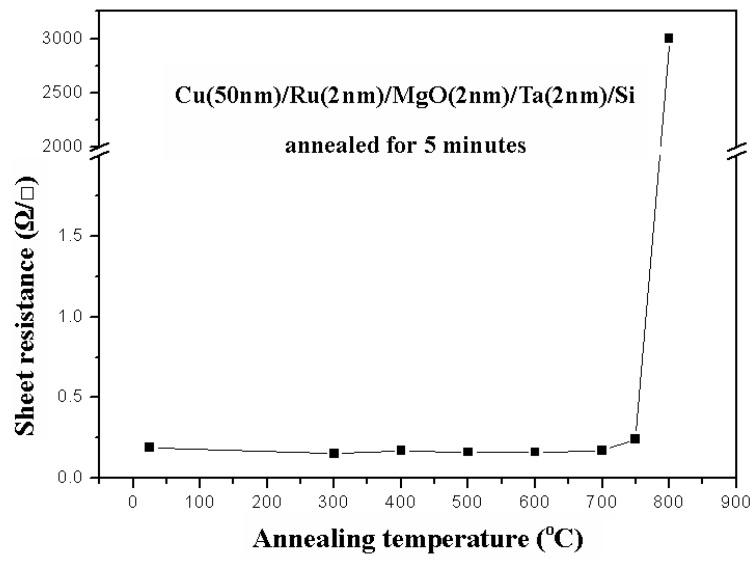
Relation between sheet resistances and annealing temperatures for a Cu (50 nm)/Ru (2 nm)/MgO (2 nm)/Ta (2 nm)/Si structure annealed for 5 min.

**Figure 9:**
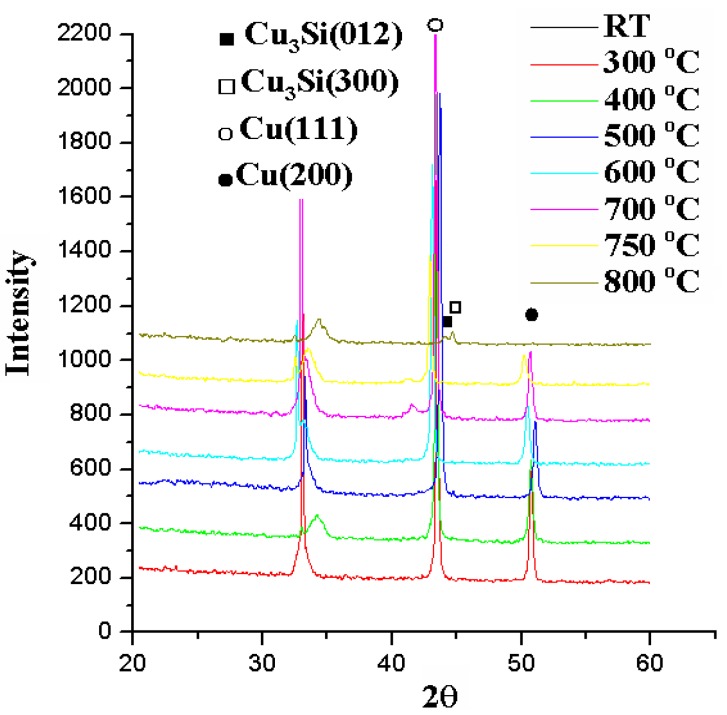
X-ray diffraction patterns for the structures of MgO (2 nm) sample annealed for 5 min at temperatures from room temperature to 800 °C.

Figure 8 shows the relation between sheet resistances and annealing temperatures for a Cu (50 nm)/Ru (2 nm)/MgO (2 nm)/Ta (2 nm)/Si structure annealed for 5 min. The sheet resistances remain at about 0.25 Ω/ϒ when annealing temperature is not higher than 750 °C, but the sheet resistance increases abruptly up to 3000 Ω/ϒ at 800 °C. The X-ray diffraction patterns for the structure of Cu (50 nm)/Ru (2 nm)/MgO (2 nm)/Ta (2 nm)/Si annealed for 5 min at temperatures from 300 to 800 °C are shown in Figure 9. The Cu_3_Si phase occurs only at annealing temperature of 800 °C.

**Figure 10:**
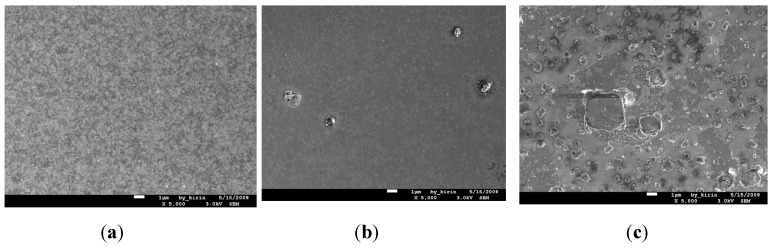
SEM views for the surface morphologies of structures of MgO (2 nm) sample annealed for 5 min at temperatures 700 °C (**a**); 750 °C (**b**); and 800 °C (**c**); respectively.

The SEM images for the surface morphologies of Cu (50 nm)/Ru (2 nm)/MgO (2 nm)/Ta (2 nm)/Si structures annealed for 5 min at 700, 750, and 800 °C are shown in Figure 10a–c, respectively. The surface of the structure annealed at 700 °C looks smooth. There are several 1 μm particles on the surface of the structure after annealing at 750 °C. Figure 10c shows there are many particles about 0.1–10 μm on the surface of the structure annealing at 800 °C.

Although the peaks of Cu_3_Si phase occur only at the X-ray diffraction pattern for the structure annealed at 800 °C, as shown in Figure 9, the Cu_3_Si particles have emerged on the surface of the structure annealed at 750 °C, as shown in Figure 10b and the sheet resistance of this structure is slightly larger than the structure annealed at 700 °C. The reason for the inconsistent phenomena may be ascribed that the amount of the Cu_3_Si phase is too small to be detected by the X-ray diffraction method.

### 2.3. Cu (50 nm)/Ru (2 nm)/MgO (1 nm)/Ta (2 nm)/Si

**Figure 11:**
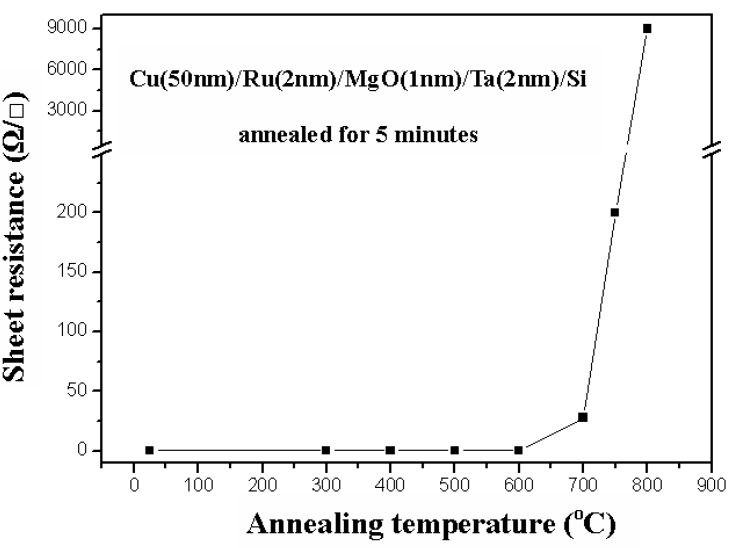
Relation between sheet resistance and annealing temperature for Cu (50 nm)/Ru (2 nm)/MgO (1 nm)/Ta (2 nm)/Si structures annealed for 5 min.

**Figure 12:**
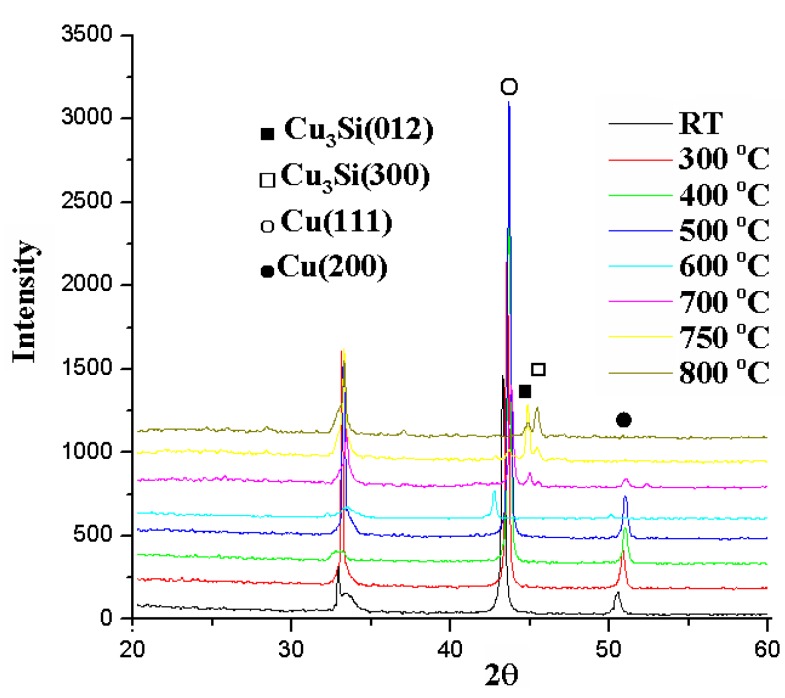
X-ray diffraction patterns for the structures of MgO (1 nm) sample annealed for 5 min at temperatures from room temperature to 800 °C.

The relation between sheet resistance and annealing temperature for the Cu (50 nm)/Ru (2 nm)/MgO (1 nm)/Ta (2 nm)/Si structures annealed for 5 min is shown in Figure 11, in which the sheet resistances are about 0.25 Ω/ϒ for the structures annealed at a temperature lower than 600 °C, about 35 Ω/ϒ for 700 °C, about 200 Ω/ϒ for 750 °C, and increases abruptly up to 9000 Ω/ϒ for 800 °C. The X-ray diffraction patterns for the Cu (50 nm)/Ru (2 nm)/MgO (1 nm)/Ta (2 nm)/Si structures annealed for 5 min at room temperature to 800 °C are shown in Figure 12, in which the Cu_3_Si phase peaks appear at annealing temperature of 700, 750, and 800 °C.

**Figure 13:**
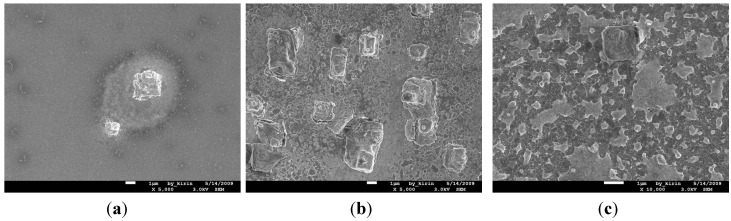
SEM views for the surface morphologies of structures of MgO (1 nm) sample annealed for 5 min at temperatures at 700 °C (**a**); 750 °C (**b**); and 800 °C (**c**); respectively.

The SEM images for the surface morphologies of the Cu (50 nm)/Ru (2 nm)/MgO (1 nm)/Ta (2 nm)/Si structures annealed for 5 min at 700 °C, 750, and 800 °C are shown in Figure 13a–c, respectively. The number of Cu_3_Si particles emerged on the surface increases with the increase of annealing temperature from 700 °C to 800 °C. It is obvious that there is a consistence among the sheet resistance, Cu_3_Si phase, and surface morphology for the structures annealed for 5 min at room temperature to 800 °C.

### 2.4. Cu (50 nm)/Ru (2 nm)/MgO (0.5 nm)/Ta (2 nm)/Si

**Figure 14:**
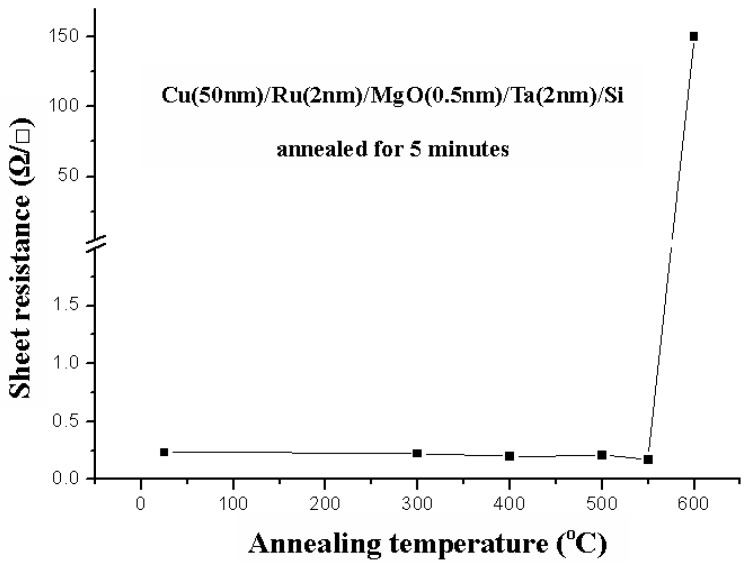
Relation between sheet resistances and annealing temperatures for the Cu (50 nm)/Ru (2 nm)/MgO (0.5 nm)/Ta (2 nm)/Si structures annealed for 5 min.

**Figure 15:**
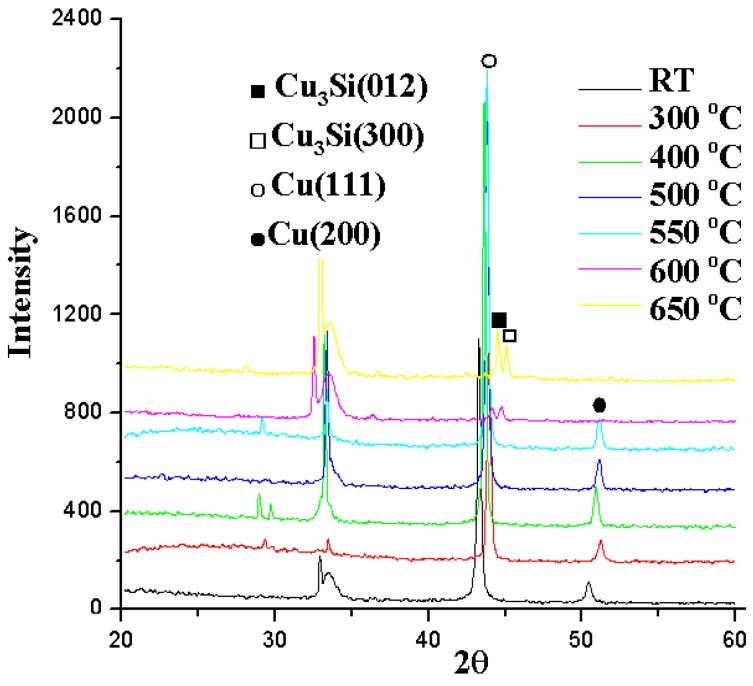
X-ray diffraction patterns for the structures of MgO (0.5 nm) sample annealed for 5 min at temperatures from room temperature to 650 °C.

Figure 14 shows the relation between sheet resistance and annealing temperature for the Cu (50 nm)/Ru (2 nm)/MgO (0.5 nm)/Ta (2 nm)/Si structures annealed for 5 min. When the annealing temperature is equal to and lower than 550 °C, the sheet resistances are about 0.25 Ω/ϒ, and when the annealing temperature is 600 °C, the sheet resistance increases to about 150 Ω/ϒ. Figure 15 shows the X-ray diffraction patterns for the structures of Cu (50 nm)/Ru (2 nm)/MgO (0.5 nm)/Ta (2 nm)/Si annealed for 5 min at a temperature from room temperature to 650 °C. It can be seen that the Cu_3_Si phase peaks emerge at 600 °C and 650 °C.

**Figure 16:**
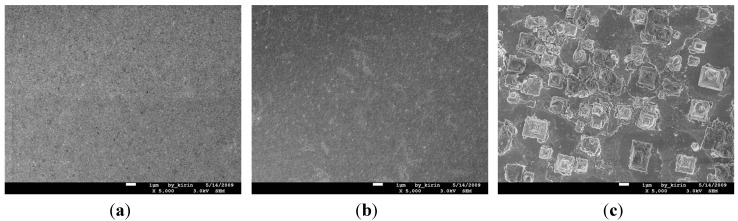
SEM images for the surface morphologies of structures of MgO (0.5 nm) sample annealed for 5 min at temperature 500 °C (**a**); 550 °C (**b**); and 600 °C (**c**), respectively.

Figure 16a–c show the SEM images for the surface morphologies of the Cu (50 nm)/Ru (2 nm)/MgO (0.5 nm)/Ta (2 nm)/Si structures annealed for 5 min at a temperature of 500, 550, and 600 °C, respectively. The surface is quite smooth in Figure 16a annealed at 500 °C, more or less rough in Figure 16b annealed at 550 °C, and has many Cu_3_Si particles in Figure 16c annealed at 600 °C. It can be seen from Figure 14, Figure 15 and Figure 16 that there is also a correspondence between the sheet resistance, Cu_3_Si phase, and surface morphology for the structures annealed for 5 min at room temperature to 600 °C.

### 2.5. Effects of MgO Layer Thicknesses and Annealing Times on the Failure of the Diffusion Barriers

Table 1 lists the maximum annealing temperatures at which the structures of Cu (50 nm)/Ru (2 nm)/MgO (0.5–3 nm)/Ta (2 nm) /Si are annealed for 5 and 30 min and still have low sheet resistance. It can be seen that the maximum annealing temperature for 5 min is 550, 600, 750, and 750 °C, and for 30 min is 500, 600, 650, and 700 °C for the structure with an MgO layer of 0.5, 1, 2, and 3 nm, respectively. The longer the annealing time is, the lower the maximum annealing temperature is, and the larger the thickness of MgO layer is, the higher the maximum annealing temperature is.

**Table 1:** Maximum annealing temperatures for various structures with low sheet resistance after annealing.

Annealing Time	Thickness of MgO Layers			
	0.5 nm	1 nm	2 nm	3 nm
5 min	550 °C	600 °C	750 °C	750 °C
30 min	500 °C	600 °C	650 °C	700 °C

## 3. Experimental Section

The substrate for the deposition of Ta, MgO, Ru, and Cu thin films is a p-type (100) Si wafer which was first cleansed by the RCA process, *i.e.*, successively dipped in an acetone solution with supersonic vibration for 15 min, in a mixture solution of H_2_SO_4_ and H_2_O_2_ (H_2_SO_4_:H_2_O_2_ = 1:1) for 10min, and in a HF solution (10%) for 2 min. After each step the Si substrate was rinsed with deionized water. The Si substrate was finally dried in N_2_ stream.

The Si substrate was then put in a sputtering chamber for the deposition of Ta, MgO, and Ru ultra-thin films. The Ta was first deposited on Si substrate in Ar gas with a working pressure of 0.36 Pa under a DC power of 100 W, therefore a plating rate of 0.051 nm/s and plating time of 39 s for a thickness of 2 nm. Then, MgO was deposited on Ta in Ar gas with a working pressure of 1 Pa under a RF power of 200 W, therefore a plating rate of 0.014 nm/s and a plating time of 36, 71, 143, and 214 s for a thickness of 0.5, 1, 2, and 3 nm, respectively. Finally, Ru was deposited on MgO in Ar gas with a working pressure of 0.36 Pa under a DC power of 100 W, therefore a plating rate of 0.063 nm/s and plating time of 32 s for a thickness of 2 nm. A Cu film about 50 nm was then deposited on the Ru film by an electroplating technique. The electroplating bath contains CuSO_4_·5H_2_O (212.5 g/L) which is the Cu^2+^ ion source. A dilute H_2_SO_4_ solution was used to adjust the pH value of electroplating bath at 2. In the electroplating process the bath temperature and the current density were controlled at 25 °C and 5 A/dm^2^, respectively.

After the Cu (50 nm)/Ru (2 nm)/MgO (0.5–3 nm)/Ta (2 nm)/Si structures were prepared, they were annealed in a rapid thermal annealing (RTA) furnace without application of external electrical fields. The annealing was carried out at a temperature from 300 to 800 °C for 5 and 30 min, respectively, to find the failure temperature and failure mechanism of Ru (2 nm)/MgO (0.5–3 nm)/Ta (2 nm) diffusion barriers. The RTA furnace was kept at 1.3 Pa pressure in the annealing process.

After the structures of Cu/Ru/MgO/Ta/Si were annealed, they were characterized by a four-point probe for their sheet resistances, by an X-ray diffractometer (MO3X-HF, Macscience Co. Ltd. Yokohama, Japan) for their crystal structures, by a scanning electron microscope (SEM, JSM-6360, JEOL, Tokyo, Japan) for their surface morphologies, by a transmission electron microscope (TEM, JEM-2010, JEOL, Tokyo, Japan) for their cross-sectional microstructures and high resolution images.

## 4. Conclusions

The ultra-thin tri-layer of Ru (2 nm)/MgO (0.5–3 nm)/Ta (2 nm) is an effective diffusion barrier against the interdiffusion between Cu film and Si substrate. The MgO, and Ta layers as deposited are amorphous. The mechanism for the failure of the diffusion barrier is that the Ru layer first became discontinuous at a high temperature and the Ta layer sequentially become discontinuous at a higher temperature, the Cu atoms then diffuse through the MgO layer and to the substrate at the discontinuities, and the Cu_3_Si phases finally form. The maximum temperature at which the structures of Cu (50 nm)/Ru (2 nm)/MgO (0.5–3 nm)/Ta (2 nm)/Si with MgO (0.5 nm), MgO (1 nm), MgO (2 nm), and MgO (3 nm) are annealed and still have low sheet resistance are 550 °C, 600 °C, 750 °C, and 750 °C for the annealing time of 5 min, respectively.
